# Female pelvic malignancy: spectrum encountered in district hospital: an aid to a general radiologist

**DOI:** 10.1186/1470-7330-15-S1-P45

**Published:** 2015-10-02

**Authors:** D Santosh, A Butler

**Affiliations:** 1 Princess Of Wales Hospital, Bridgend, UK

## Purpose

Initial diagnosis and staging of gynaecological malignancy is often the remit of the local general radiologist. The “incidentaloma” is not infrequently encountered on pelvic imaging undertaken for other indications. It can be daunting to those without a solid knowledge of the female pelvis.

## Learning objectives

To recognise the spectrum of female pelvic malignancy and appreciate relevant incidental findings.

To identify the pearls and pitfalls of diagnosis, understand important points in staging and restaging disease.

To identify potential complications of both tumour and treatment and discuss imaging strategies in suspected local recurrence.

## Content organisation

We provide an overview of imaging modalities used, review pertinent anatomy and cover the spectrum of malignancy specific to the female pelvis, incorporating common and uncommon tumours. Emphasis is placed on imaging features that are important in treatment planning and evaluating prognosis.

## Summary

This exhibit will enhance the confidence of general radiologist in a district hospital setting when encountering potential female pelvic malignancy at initial diagnosis or when assessing for complications of tumour or treatment in the acute setting.

